# Abscisic acid transcriptomic signaling varies with grapevine organ

**DOI:** 10.1186/s12870-016-0763-y

**Published:** 2016-03-22

**Authors:** Supakan Rattanakon, Ryan Ghan, Gregory A. Gambetta, Laurent G. Deluc, Karen A. Schlauch, Grant R. Cramer

**Affiliations:** Department of Biochemistry and Molecular Biology, University of Nevada, Reno, NV 89557 USA; Bordeaux Sciences Agro, Institut des Sciences de la Vigne et du Vin (ISVV), EGFV, UMR 1287, F-33140 Villenave d’Ornon, France; Department of Horticulture, Oregon State University, Corvallis, OR 97331 USA

**Keywords:** Abscisic acid signaling, Organ-specificity, Transcriptomics, *Vitis vinifera* L

## Abstract

**Background:**

Abscisic acid (ABA) regulates various developmental processes and stress responses over both short (i.e. hours or days) and longer (i.e. months or seasons) time frames. To elucidate the transcriptional regulation of early responses of grapevine (*Vitis vinifera*) responding to ABA, different organs of grape (berries, shoot tips, leaves, roots and cell cultures) were treated with 10 μM (*S*)-(+)-ABA for 2 h. NimbleGen whole genome microarrays of *Vitis vinifera* were used to determine the effects of ABA on organ-specific mRNA expression patterns.

**Results:**

Transcriptomic analysis revealed 839 genes whose transcript abundances varied significantly in a specific organ in response to ABA treatment. No single gene exhibited the same changes in transcript abundance across all organs in response to ABA. The biochemical pathways affected by ABA were identified using the Cytoscape program with the BiNGO plug-in software. The results indicated that these 839 genes were involved in several biological processes such as flavonoid metabolism, response to reactive oxygen species, response to light, and response to temperature stimulus. ABA affected ion and water transporters, particularly in the root. The protein amino acid phosphorylation process was significantly overrepresented in shoot tips and roots treated with ABA. ABA affected mRNA abundance of genes (*CYP707As, UGTs,* and *PP2Cs*) associated with ABA degradation, conjugation, and the ABA signaling pathway. ABA also significantly affected the expression of several transcription factors (e.g. AP2/ERF, MYC/MYB, and bZIP/AREB). The greatest number of significantly differentially expressed genes was observed in the roots followed by cell cultures, leaves, berries, and shoot tips, respectively. Each organ had a unique set of gene responses to ABA.

**Conclusions:**

This study examined the short-term effects of ABA on different organs of grapevine. The responses of each organ were unique indicating that ABA signaling varies with the organ. Understanding the ABA responses in an organ-specific manner is crucial to fully understand hormone action and plant responses to water deficit.

**Electronic supplementary material:**

The online version of this article (doi:10.1186/s12870-016-0763-y) contains supplementary material, which is available to authorized users.

## Background

Decreasing water resources and increasing global warming have the potential to reduce food production in the future [1, 2]. Abiotic stresses such as drought, cold and salinity have large impacts on plant growth and development leading to a loss of production and reduced crop quality, which results in the loss of hundreds of millions of dollars each year. Changes in climate that lead to an increase in the frequency and magnitude of drought stress will increase a crop’s dependence on irrigation to maintain productivity.

Grapevine (*Vitis vinifera* L.) is one of the most economically important fruit crops affected by abiotic stresses. Grapes have a multi-billion dollar impact on the economy, as well as having health benefits, such as providing nutrients and antioxidants [3]. Cabernet Sauvignon is one of the most well known red wine grapes in the world and is widely cultivated in water-limited areas of the world (e.g. California, Chile and South Australia) where production is highly dependent on irrigation. Grape yields are influenced by plant water status and water stress can lead to decreases in grape production and affect wine quality [4, 5].

The plant hormone abscisic acid (ABA) plays a crucial role in responding to a variety of environmental stresses such as drought, salinity and chilling stress [1, 6] and has essential functions involved in plant growth and development, including seed germination, seed dormancy and bud dormancy [7–9]. ABA has an important role in vegetative tissues in conserving water loss by closing stomata and reducing the leaf surface area. ABA is increased in response to water deficit in grapevine leaves, xylem sap, and berries [10, 11] and water deficit affects a large number of transcripts involved in ABA metabolism [10–13].

In the past years, ABA signal transduction has been extensively studied at the molecular level [6, 14–17]. As a result, numerous secondary messengers associated with ABA signaling such as calcium (Ca^2+^), reactive oxygen species (ROS), and nitric oxide (NO) were identified. An ABA model of action utilizing PYR/PYL/RCAR receptors, type 2C protein phosphatases (PP2C) and sucrose non-fermenting-1 (SNF1)-related protein kinase 2 (SnRK2) was proposed and validated [18–20]. The soluble PYR/PYL/RCAR receptors function at the apex of a negative regulatory pathway to directly regulate PP2C, which in turn negatively regulates SnRK2. SnRK2 is auto-phosphorylated and then phosphorylates other transcription factors (TFs), such as members of the bZIP/ABRE, NAC, MYC/MYB, and AP2/ERF TF families. However, their interactions in the framework of an ABA signaling network remain to be clarified.

The first step of *de novo* ABA biosynthesis in response to stimuli occurs in the plastid and in the final step, ABA-aldehyde is converted to ABA in the cytosol [21]. ABA is catabolized by ABA 8′-hydroxylases and conjugated by ABA glucosyltransferases. ABA-glucose ester (ABA-GE) levels in leaves were shown to be relatively constant under normal conditions and substantially increase during drought stress [22]. ABA-GE is a transport and storage form of ABA, which is critical for ABA homeostasis [23, 24]. There are at least two different plasma membrane-localized ABA transporters; ATP-BINDING CASSETTE G25 (ABCG25) is a transporter for ABA efflux from vascular tissue [25] and ABCG40 is responsible for ABA transport into guard cells in *Arabidopsis* [26].

Plants contain multiple organs that have specific physiological functions with unique gene expression patterns during different developmental stages and stress responses. For example, leaves specialize in photosynthesis and roots specialize in ion and water transport. ABA affects gene expression differently in seeds and seedlings of *Arabidopsis* [7]; however little is known about other organs, particularly in a woody perennial fruit crop like grapevine. Physiological responses to ABA differ in different organs and cells types such as roots, shoots and guard cells. We hypothesize that ABA signaling will differ in different organs as well. Investigations of ABA signaling in different organs will improve our understanding of plant responses to osmotic stress and plant development.

This study focuses on ABA signaling, investigating the down-stream transcriptional gene expression in different organs: roots shoot tips, mature leaves, berries and cell culture (meristem-like cells). The work presented here elucidates the effect of ABA on the transcription of genes involved in ABA biosynthesis, degradation, conjugation, transport, signaling pathways and metabolic pathways in different organs of grapevine.

## Results

### Transcriptomic analysis of grapevine shows organ-specific change in response to ABA

**Fig. 1:**
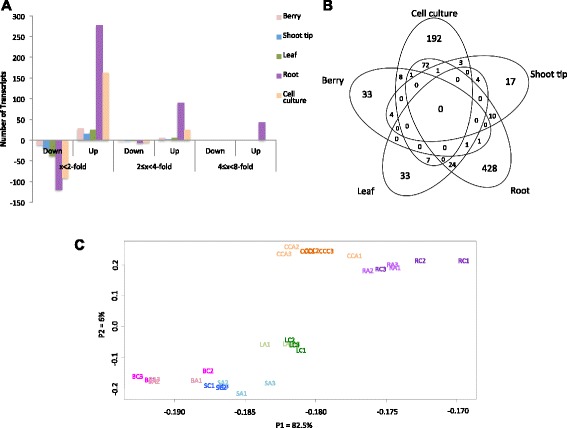
Gene expression of control and ABA treated-plants. **a** Numbers of significantly differentially expressed genes induced by ABA. **b** Venn diagram showing the overlap of significantly differentially expressed genes between control and ABA treatment in berries, shoots, roots, leaves and cell cultures. **c** Principal component analysis of berries (B), shoot tips (S), leaves (L), roots (R), and cell cultures (CC). C = Control; A = ABA treated. The following numbers refers to an experimental replicate sample

Five different organs (berries, shoot tips, leaves, roots and cell cultures) of Cabernet Sauvignon were directly exposed to 10 μM ABA for 2 h, except the leaf samples, which were harvested from vines that had their roots treated aeroponically. Although anatomically incorrect, we refer to cell cultures as artificial organs representing meristem-like functions in this paper. The NimbleGen *Vitis* whole genome microarray was used to test our hypothesis that different organs had different ABA signaling responses. A two-way ANOVA identified 545 genes with a statistically significant treatment effect, and 644 genes with a statistically significant ABA treatment and organ interaction (adjusted *p*-values with *p* ≤ 0.05, upon adjustment for the false discovery rate; FDR) (Additional file 1). The term “significant(ly)” will mean “statistically significant” at a p-value at or below 0.05 throughout this paper. A post-hoc Tukey test was performed to identify significant treatment effects among each organ; there were 839 significant (*p* ≤ 0.05) differentially expressed genes (DEGs) based upon the Tukey post-hoc test between control and ABA treatment in at least one organ (Additional file 2). From the post-hoc test, the root had 538 DEGs, which is the largest number of genes in response to ABA, while the shoot tip had the lowest number with 39 genes (Fig. 1a). To show the distribution of the DEGs between control and ABA treatment among berries, shoot tips, leaves, roots, and cell cultures, a five-way Venn diagram was created (Fig. 1b). Roots and cell cultures had the most overlap with 74 genes responding in common to ABA. Our hypothesis was confirmed, there was not a single gene whose transcript abundance changed in common with all organs; the response to ABA was organ-specific. Further supporting our hypothesis, principal component analysis (PCA) revealed distinct differences among the different organs and treatments within the transcriptomic data (Fig. 1c). Principal component 1 and 2 (82.5 and 6 %, respectively) show the overall variance of transcription expression values. Grape organs were clearly separated from each other on the first principal component; however, on the second principal component, berries and shoot tips were separated from leaves as well as cell cultures and roots.

### ABA affects ABA biosynthesis, degradation, conjugation, transport, signaling and metabolic pathways

**Fig. 2:**
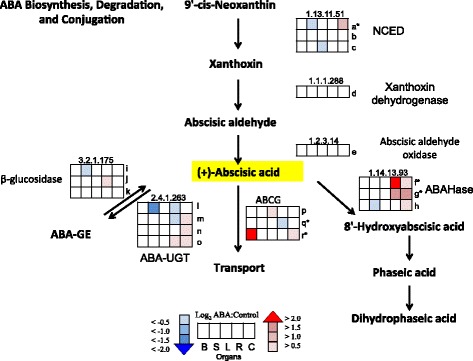
ABA Biosynthesis, Degradation, and Conjugation. The profiles of the log_2_ ratio (ABA/Control) of the transcript abundance of the genes (probesets) matched to each enzyme in the ABA metabolic pathway are shown as heat maps in the boxes. *Boxes* from left to right are berries (B), shoot tips (S), leaves (L), roots (R), and cell cultures (C), respectively. EC numbers and all abbreviations are: 1.13.11.51 (NCED: nine-cis-epoxycarotenoid dioxygenase), 1.1.1.288 (xanthoxin dehydrogenase), 1.2.3.14 (AAO3: abscisic aldehyde oxidase), 1.14.13.93 (ABAHase: ABA 8′-hydroxylase), 2.4.1.263 (ABA-UGT: abscisate beta-glucosyltransferase), 3.2.1.175 (β-D-glucopyranosyl abscisate β-glucosidase), ABA-GE: ABA-glucose ester, ABCG: ATP-binding cassette subfamily G. All current V1 IDs for a-r found in Additional file 3 are: a = EnsemblPlants:VIT_19s0093g00550, b = EnsemblPlants:VIT_10s0003g03750, c = EnsemblPlants:VIT_05s0051g00670, d = EnsemblPlants:VIT_13s0019g01010, e = EnsemblPlants:VIT_06s0009g00770, f = EnsemblPlants:VIT_06s0004g05050, g = EnsemblPlants:VIT_03s0063g00380, h = EnsemblPlants:VIT_02s0087g00710, i = EnsemblPlants:VIT_06s0004g01430, j = EnsemblPlants:VIT_01s0011g00760, k = EnsemblPlants:VIT_17s0000g02680, l = EnsemblPlants:VIT_12s0034g00160, m = EnsemblPlants:VIT_12s0055g00020, n = EnsemblPlants:VIT_12s0055g00030, o = EnsemblPlants:VIT_03s0063g00040, p = EnsemblPlants:VIT_18s0072g01220, q = EnsemblPlants:VIT_09s0002g05400, r = EnsemblPlants:VIT_18s0166g00080. * indicate the significant genes from the Tukey Post-Hoc test (*p* ≤ 0.05)

ABA had significant effects on the transcript abundance of genes involved in ABA metabolism (Fig. 2; additional details of the gene annotations and expression values are provided in Additional file 3). Carotenoids are the precursors for ABA biosynthesis. ABA biosynthesis is often controlled by the rate-limiting step, nine-cis epoxycarotenoid dioxygenase (NCED) [27, 28]. The transcript abundance of *VviNCED3* [EnsemblPlants:VIT_19s0093g00550] significantly increased in cell cultures. Note this gene was originally named *VvNCED1* [10], but following the recommendations of the International Grape Genome Program Supernomenclature Committee [29], we have named this gene *VviNCED3*, because its closest ortholog in Arabidopsis is *AtNCED3*.

The transcript abundances of genes encoding enzymes in the ABA biosynthesis pathway before the rate-limiting step did not change significantly in response to exogenous ABA in most organs; the exception was a significant increase in transcript abundance for β-carotene hydroxylase [EnsemblPlants:VIT_16s0050g01090] in cell culture (Additional file 3). The transcript abundance of xanthoxin dehydrogenase and abscisic aldehyde oxidase genes, two steps involved in ABA biosynthesis after NCED, was not significantly affected by ABA.

The ABA level in cells is highly regulated by degradation and conjugation processes. ABA is metabolized to 8′-hydroxyabscisic acid by ABA 8’-hydroxylase (ABAHase) and conjugated to ABA-GE by ABA UDP-glucosyltransferase (ABA-UGT). The relative expression of genes involved in ABA catabolism, *CYP707As* [EnsemblPlants:VIT_06s0004g05050 and EnsemblPlants:VIT_03s0063g00380] [30] were significantly increased in roots and cell cultures in response to ABA. For ABA conjugation, the transcript abundance of the *UGT* genes was decreased in the shoot tips and roots but increased in cell cultures. The transcript abundance of an ABA transporter, *VviABCG40* [EnsemblPlants:VIT_09s0002g05560], significantly decreased in roots. Another transcript annotated as an ABC transporter G member 22-like [EnsemblPlants:VIT_18s0166g00080] may also be involved in ABA transport as it is a paralog of the *Vitis* ortholog [EnsemblPlants:VIT_08s0032g00790] of *AtABCG22* (At5g06530); its transcript abundance increased significantly in berries in response to ABA.

**Fig. 3:**
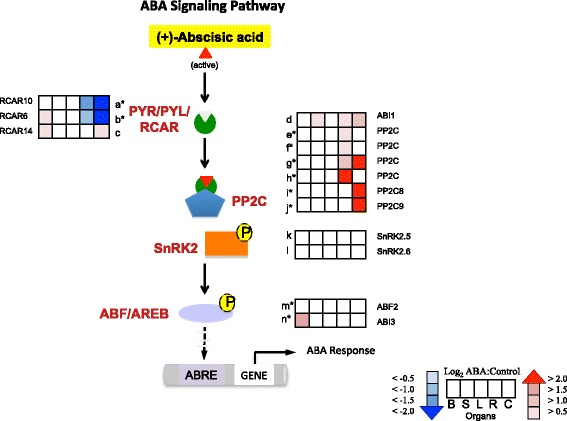
ABA Signaling Pathway. The profiles of the log_2_ ratio (ABA/Control) of the transcript abundance of the genes (probe sets) matched to each enzyme in the ABA signaling pathway are shown as heat maps in the boxes. *Boxes* from left to right are berries (B), shoot tips (S), leaves (L), roots (R), and cell cultures (C), respectively. All abbreviations are: PYR/PYL/RCAR: pyrabactin resistance/PYR-like/regulatory components of ABA receptor, PP2C: protein phosphatase 2C, SnRK2: sucrose non-fermenting 1-related protein kinase 2, AREB/ABF: ABA-responsive element binding protein/ABRE binding factor, ABRE: ABA-responsive element. All current V1 IDs for a-m found in Additional file 3 are: a = EnsemblPlants:VIT_08s0058g00470, b = EnsemblPlants:VIT_13s0067g01940, c = EnsemblPlants:VIT_04s0008g00890, d = EnsemblPlants:VIT_11s0016g03180, e = EnsemblPlants:VIT_16s0050g02570, f = EnsemblPlants:VIT_06s0004g06840, g = EnsemblPlants:VIT_16s0050g02680, h = EnsemblPlants:VIT_00s0179g00140, i = EnsemblPlants:VIT_13s0019g02200, j = EnsemblPlants:VIT_06s0004g05460, k = EnsemblPlants:VIT_07s0197g00080, l = EnsemblPlants:VIT_03s0063g01080, m = EnsemblPlants:VIT_18s0001g10450, n = EnsemblPlants:VIT_07s0005g05400. * indicate the significant genes from the Tukey Post-Hoc test (*p* ≤ 0.05)

Heat maps displaying the gene expression profile of core components of the classic ABA signaling pathway (PYR/PYL/RCAR, PP2C, SnRK2, and ABA-responsive element binding transcription factors) are shown in Fig. 3 and Additional file 3. The transcript abundance of the genes encoding PYL12/RCAR6 [EnsemblPlants:VIT_13s0067g01940] and PYL4/RCAR10 [EnsemblPlants:VIT_08s0058g00470] decreased significantly in roots and cell cultures in the presence of ABA. In contrast, the relative gene expression of PP2Cs increased significantly in roots [EnsemblPlants:VIT_16s0050g02570 and EnsemblPlants:VIT_06s0004g06840] and cell cultures [EnsemblPlants:VIT_13s0019g02200 and EnsemblPlants:VIT_06s0004g05460] in response to ABA. There were no significant changes in gene expression of SnRK2s following exposure to ABA. A significant increase in transcript abundance of *VviABF2* [EnsemblPlants:VIT_18s0001g10450] was found in roots.

**Fig. 4:**
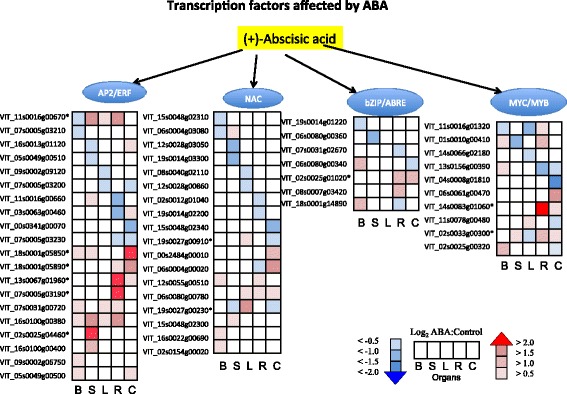
Transcription factors affected by ABA. The profiles of the log_2_ ratio (ABA/Control) of the transcript abundance of the genes (probe sets) matched to each transcription factors in the ABA signaling pathway are shown as heat maps in the boxes. *Boxes* from left to right are berries (B), shoot tips (S), leaves (L), roots (R), and cell cultures (C), respectively. * indicate the significant genes from the Tukey Post-Hoc test (*p* ≤ 0.05)

Both ABA-dependent and -independent transcription factors such as members of the AP2/ERF, NAC, bZIP/ABRE, and MYC/MYB families were affected by ABA (Fig. 4; Additional file 3). Interestingly, the greatest change in transcript abundance of transcription factors was found in the AP2/ERF family, with 20 members changing in their transcript abundance with statistical significance. The highest increase in transcript abundance induced by ABA of the AP2/ERF TF superfamily was *VviDREB2H* [EnsemblPlants:VIT_13s0067g01960]; the response of *VviDREB2H* was root specific. In the NAC TF family, there were 18 DEGs. A transcript encoding a gene ortholog to *VviNAC1* [EnsemblPlants:VIT_19s0027g00230] underwent a significant increase in transcript abundance in leaves. There was a slight change in the transcript abundances of seven members of the bZIP/ABRE TF family. One DEG of the bZIP/ABRE TF family was *VviGBF3* [EnsemblPlants:VIT_02s0025g01020], which was found to be significantly upregulated in roots and cell cultures. There is no ortholog of this gene in Arabidopsis; its function is unknown. For the MYC/MYB TF family, there were 10 DEGs. Most notably, *VviMYB121* [EnsemblPlants:VIT_14s0083g01060] exhibited the greatest significant increase in transcript abundance in roots.

**Fig. 5:**
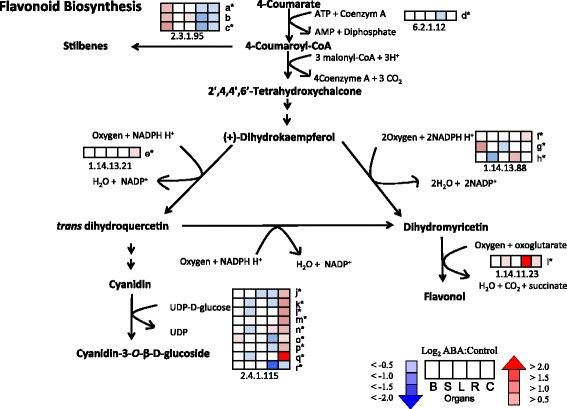
Flavonoid biosynthesis pathway. The profiles of the log2 ratio (ABA/Control) of the transcript abundance of the genes (probe sets) matched to each enzyme in the flavonoid biosynthesis pathway are shown as heat maps in the boxes. *Boxes* from left to right are berries (B), shoot tips (S), leaves (L), roots (R), and cell cultures (C), respectively. EC numbers are: 2.3.1.95 (stilbene synthase), 6.2.1.12 (4-coumarate-CoA ligase 2), 1.14.13.21 (flavonoid 3’-hydroxylase), 1.14.13.88 (flavonoid 3′,5′-hydroxylase), 1.14.11.23 (flavonol synthase), 2.4.1.115 (anthocyanidin 3-O-glucosyltransferase). All current V1 IDs for a-r found in Additional file 3 are: a = [EnsemblPlants:VIT_16s0100g00860], b = [EnsemblPlants:VIT_10s0042g00910], c = [EnsemblPlants:VIT_10s0042g00870], d = [EnsemblPlants:VIT_17s0000g01790], e = [EnsemblPlants:VIT_17s0000g07200], f = [EnsemblPlants:VIT_05s0094g01190], g = [EnsemblPlants:VIT_07s0031g01570], h = [EnsemblPlants:VIT_18s0001g00590], i = [EnsemblPlants:VIT_18s0001g03510], j = [EnsemblPlants:VIT_12s0034g00030], k = [EnsemblPlants:VIT_12s0034g00060], l = [EnsemblPlants:VIT_12s0034g00130], m = [EnsemblPlants:VIT_12s0034g00140], n = [EnsemblPlants:VIT_12s0034g01120], o = [EnsemblPlants:VIT_12s0055g00290], p = [EnsemblPlants:VIT_17s0000g04750], q = [EnsemblPlants:VIT_17s0000g04760], r = [EnsemblPlants:VIT_17s0000g07100. * indicate the significant genes from the Tukey Post-Hoc test (*p* ≤ 0.05)

The transcript abundance of genes in several metabolic pathways was affected by exogenous ABA, especially the flavonoid biosynthesis pathway (Fig. 5; Additional file 3). Genes encoding stilbene synthase [STS: EnsemblPlants:VIT_16s0100g00860 and EnsemblPlants:VIT_10s0042g00870] significantly increased in transcript abundance in response to ABA treatment in berries but decreased in roots and cell cultures. On the contrary, genes encoding flavonol synthase [EnsemblPlants:VIT_18s0001g03510] and flavonoid 3’-hydroxylase [EnsemblPlants:VIT_17s0000g07200] were significantly increased in roots in response to ABA. Interestingly, the transcript abundance of many genes encoding anthocyanidin 3-O-glucosyltransferase was significantly increased in response to ABA only in roots, while other organs showed decreasing or unchanging mRNA abundance.

### Differential gene expression responses of organs to ABA

ABA affected many biological processes of the gene ontology (GO) functional categories; statistically significant DEGs were involved in protein folding and response to heat, hydrogen peroxide, high light, and temperature (Additional file 4). However, each plant organ had a different response to ABA. The individual responses of each organ are summarized below:

#### Berry

There were 33 genes that showed organ-specific gene expression responses to ABA in berries (Fig. 1b; Additional file 2). The gene with the highest increase in transcript abundance to ABA was *VviABCG22-like* [EnsemblPlants:VIT_18s0166g00080]; while the largest decrease in transcript abundance was a gene encoding a glycine-rich cell wall structural protein [EnsemblPlants:VIT_05s0077g00900]. The gene encoding the transcription factor (TF) with the highest transcript abundance in response to ABA in berries was *abscisic acid insensitive 3-like* [*VviABI3*: EnsemblPlants:VIT_07s0005g05400]. ABI3 is a B3-domain transcription factor that is a part of the core ABA signaling network [31]. The transcript abundance of a *peroxidase* gene [EnsemblPlants:VIT_04s0008g07040] involved in porphyrin and chlorophyll metabolism significantly decreased in response to ABA. Another transporter that significantly increased in transcript abundance was a vacuolar amino acid transporter [EnsemblPlants:VIT_19s0027g01870]. The transcript abundance of a *GABAT* (γ-aminobutyric acid-transaminase) gene [EnsemblPlants:VIT_03s0017g01720] increased significantly, while the expression of *AST* [aspartate aminotransferase: EnsemblPlants:VIT_05s0020g03410] decreased significantly, which may cause an increase of succinate followed by an increase of malate. Transcript abundances of genes encoding enzymes involving flavonoid biosynthesis such as *STS* [EnsemblPlants:VIT_16s0100g00860 and EnsemblPlants:VIT_10s0042g00870] significantly increased only in the berry.

#### Shoot tip

Genes with significantly decreased transcript abundance in the ABA treatment of shoot tips that had significantly overrepresented GO categories were involved in protein amino acid phosphorylation (Additional file 4) such as a clavata1-like receptor kinase [*VviCLV1;* EnsemblPlants:VIT_04S0008G00350], receptor kinase homolog LRK14-like [EnsemblPlants:VIT_16s0098g00080], and malectin/receptor-like protein kinase-like [EnsemblPlants:VIT_16s0039g01260]. There were 17 genes that showed organ-specific gene expression responses to ABA (Fig. 1b; Additional file 2). The most highly expressed genes in response to ABA were the genes in the AP2/ERF TF family. A *VviDDF2* [EnsemblPlants:VIT_02s0025g04460] was the gene with the greatest increase in transcript abundance and was significantly changed in the shoot tips. A *VviORA47* [EnsemblPlants:VIT_11s0016g00670] was also significantly increased in response to ABA. The genes with the largest decrease in transcript abundance in response to ABA were involved in secondary cell wall biosynthesis [fasciclin-like arabinogalactan protein genes: *VviFLA11*; EnsemblPlants:VIT_08s0040g01990]. Another gene that is also involved in cell wall biosynthesis [xyloglucosyltransferase: EnsemblPlants:VIT_14s0060g01670] was significantly decreased in transcript abundance in response to ABA. This result indicates that there may be an inhibition of shoot growth in response to ABA.

#### Leaf

There were 33 genes that showed leaf organ-specific gene expression in response to ABA (Fig. 1b; Additional file 2). The greatest increase of transcript abundance of TFs was *VviNAC1* [EnsemblPlants:VIT_19s0027g00230]. The gene with the largest decrease in transcript abundance in response to ABA was a gene encoding ent-copalyl diphosphate synthase [EnsemblPlants:VIT_07s0151g01070], which is involved in gibberellic acid (GA) biosynthesis. The transcript abundance of ent-copalyl diphosphate synthase (At4G02780) in *Arabidopsis* also decreased in response to ABA [32]. Most genes with significantly increased transcript abundance in leaves were involved in protein folding. There were also genes encoding (E,E)-alpha-farnesene synthases [EnsemblPlants:VIT_00s0361g00060 and EnsemblPlants:VIT_00s0392g00030] that significantly increased in transcript abundance in response to ABA in leaves but significantly decreased in shoot tips. (E,E)-alpha-farnesene synthases are involved in terpenoid biosynthesis. Some genes with statistically significant decreased transcript abundance, such as *epidermal patterning factor-like protein 3* [EnsemblPlants:VIT_09s0002g01700] and *TAPETUM 1* [EnsemblPlants:VIT_01s0137g00030] have roles in leaf morphogenesis and bract formation. Furthermore, there were genes with significantly decreased transcript abundance related to transport such as an aquaporin *GAMMA-TIP3* [EnsemblPlants:VIT_06s0061g00730] and an ortholog of ABC transporter member 5, *AtMRP5* [EnsemblPlants:VIT_07s0005g04460], which is an inositol hexakisphosphate transporter involved in ABA signaling and regulation of guard cells [33].

#### Root

Roots have the largest number of genes responding to ABA and there were a number of GO categories that were significantly overrepresented (Additional file 4). The main Biological Process GO categories for genes with a significant increase in transcript abundance in root in response to ABA was response to stimuli such as light, heat, and reactive oxygen species. The molecular function of (+)-abscisic acid 8′-hydroxylase activity was significantly overrepresented for genes with a significant increase in response to ABA. For genes with a significant decrease in transcript abundance to ABA, many of them were involved in protein amino acid phosphorylation. The transcript abundance of *VviMYB121* [EnsemblPlants:VIT_14s0083g01060] and *VviDREB2H* [EnsemblPlants:VIT_13s0067g01960] was significantly upregulated only in the root (Additional file 2). These two transcription factors have been found to respond to abiotic stress. qPCR results also confirmed that the transcript abundance of *VviMYB121* and *VviDREB2H* increased in response to ABA in roots (Additional file 5). The genes with the largest decrease in transcript abundance in response to ABA in the root were two pectinesterases [EnsemblPlants:VIT_06s0009g02560 and EnsemblPlants:VIT_06s0009g02570] (Additional file 2), pectin-degrading enzymes that are involved in cell wall metabolism. The transcript abundance of a number of heat shock protein genes increased in the roots. The transcript abundance of genes encoding glucosyltransferase enzymes [EnsemblPlants:VIT_05s0062g00310, EnsemblPlants:VIT_05s0062g00300, and EnsemblPlants:VIT_05s0062g00340] was significantly downregulated by ABA in the root. Interestingly, there were auxin-, anthocyanin-, and cytokinin- glucosyltransferases DEGs whose transcript abundance was decreased by ABA only in roots. Several genes involved in transport were significantly increased in roots such as *VviABCG33* [EnsemblPlants:VIT_14s0060g00470] an ortholog of *AtPDR5*, the aquaporin *GAMMA-TIP3* [EnsemblPlants:VIT_06s0061g00730], and a sulfate transporter [EnsemblPlants:VIT_05s0020g03970]. Note that the same aquaporin was downregulated in the leaf by ABA (see above).

#### Cell culture

The GO categories that were significantly overrepresented in cell cultures of up-regulated genes in response to ABA were involved in hormone stimulus (Additional file 4). In addition, there were a large number of genes with increased transcript abundance that were associated with xyloglucosyltransferase activity such as EnsemblPlants:VIT_11S0052G01190, EnsemblPlants:VIT_11S0052G01330, EnsemblPlants:VIT_11S0052G01220, and EnsemblPlants:VIT_11S0052G01300. The TF that had the highest increase in transcript abundance in response to ABA in cell cultures was an ortholog [EnsemblPlants:VIT_18s0001g05850; Fig. 4) of *AtERF022*, which is involved in somatic embryogenesis and ethylene signaling [34]. The gene with the largest decrease in transcript abundance in response to ABA was the ABA receptor *PYL12/RCAR6* [EnsemblPlants:VIT_13s0067g01940] (Additional file 5). This result is consistent with the high expression of a *PP2C* [EnsemblPlants:VIT_06s0004g05460], which is part of a negative feedback loop in the ABA signaling pathway. Lignin-forming anionic peroxidase genes were also decreased in transcript abundance by ABA [EnsemblPlants:VIT_01s0010g01920, EnsemblPlants:VIT_01s0010g02000 and EnsemblPlants:VIT_01s0010g02020]. Down-regulation of an anionic peroxidase alters both lignin content and composition [35]. The gene with the highest increase in transcript abundance was a *U-box domain-containing protein 19* [EnsemblPlants:VIT_17s0000g08080] that may have a U-box type E3 ubiquitin ligase function (Additional file 5).

## Discussion

Water deficit alters the metabolic homeostasis of plants [11, 36–38]. Plants reduce water loss by closing stomata and decreasing photosynthesis, which can be triggered by ABA [13]. ABA can be synthesized in all cells and organs [39]. A root organ senses the soil water availability, synthesizes *de novo* ABA and transports ABA via the xylem under mild water deficits [40]. However, it is still uncertain the degree to which ABA is transported from the root to different parts of the plant via the xylem sap or how much ABA is directly synthesized in leaves or guard cells, subsequently causing stomatal closure [8, 9, 40].

### ABA signaling varies with the organ

Our microarray data clearly support our hypothesis that different organs had different ABA signaling responses. No single DEG was affected in the same manner in all organs. In our study, equal concentrations of ABA were applied to the organs (except for leaves which only responded to ABA transport from the ABA applied to the root); the larger change in gene expression found in the root (Fig. 1a) may be due to a higher sensitivity for ABA in the root, however, we cannot rule out the possibility that there were differences in ABA uptake into the organs. Furthermore, with time or higher ABA concentrations, more genes may have had similar expression patterns. Nevertheless, the results support differences in ABA sensitivity and signaling.

Another contributing factor to the variation in ABA response could be the presence of other interacting signals or epigenetic regulation [41, 42]. ABA is known to interact or have crosstalk with other plant hormones and nutrients such as sugars [1, 6, 31]. Critical processes including seed germination, fruit development, root and shoot growth have been used to understand the role of hormone crosstalk in protein activities and gene expression. It is a major challenge to decipher the intricate complexity of these multiple signals in the regulation of gene expression. Our data provide direct transcriptional evidence of ABA action that differs from one organ to another. This indicates the involvement of other “signaling partners” along with ABA that are essential to coordinate gene expression. Future experiments will be needed to identify what are the major factors that may interact with ABA in order to explain the different responses of ABA in the organs studied here.

### ABA effects on ABA metabolism

Our data indicate that ABA triggered feedback loops on ABA metabolism and signaling and these responses differed among the organs. Multiple studies showed that *NCED* genes (nine-cis-epoxycarotenoid dioxygenase) were induced strongly by drought-stressed conditions in grape, *Arabidopsis*, maize, tomato, bean, cowpea and avocado [10, 12, 43–47]. In our study, the relative expression of *VviNCED3* was significantly increased in cell culture, but not other organs. The transcript abundance of genes involved in ABA degradation and conjugation was highly increased (Fig. 2). This is consistent with evidence from several studies that ABA negatively regulates its own accumulation, in part through activation of catabolic enzymes [8, 48, 49]. In the ABA signaling pathway, the transcript abundance of *PYR/PYL/RCAR* significantly decreased and the transcript abundance of *PP2Cs* significantly increased in roots and cell culture (Fig. 3). The high level of ABA from stress conditions can induce the expression of *PP2Cs* [50]*.* For ABA transport, an ABC transporter was found to be able to transport ABA from the cytoplasm to the vacuole in order to control the level of ABA in the cytosol [24]. The relatively high expression of *VviABCG22-like* in berries indicates that there may be ABA transport into cells [51]. In contrast, the significant decrease in transcript abundance of *VviABCG40* in cell cultures may lessen ABA levels in cells, which corresponds to the negative-feedback response found in cell cultures. Overall, these results indicate that there is a negative-feedback loop from the increasing ABA concentration to balance ABA action [52].

### Biochemical effects of ABA on berry ripening

In plant growth and development, the ABA level increases during grape berry ripening; possibly independent of osmotic stress [11, 53, 54]. Processes involved during grape berry ripening include: fruit softening, sugar accumulation, organic acid reduction, and increases in potassium level and phenolic compounds [55]. Our study is consistent with these studies that ABA is involved in grape berry ripening processes. The significant increase in transcript abundance of *VviABI3* in berries was found during the lag phase of berry ripening [56] indicating a relationship between ABA and grape ripening. A glycine-rich protein was found in our microarray analyses whose ortholog is involved in cell wall biogenesis and degradation in *Arabidopsis* (At3g17050). This result is consistent with the hypothesis that ABA regulates genes involved in cell wall modification during the ripening processes contributing to fruit softening. The relative expression of genes involved in malate biosynthesis such as *GABAT* and *AST* increased. GABA metabolism is able to contribute to the activity of both the TCA cycle and the respiratory electron transfer chain by generating succinate and NADH through SSADH activity. It is consistent with a low expression of an aspartate aminotransferase gene, which catalyzes oxaloacetate to aspartate leading to an increase of malate level. Moreover, ABA enhances the production of phenolic compounds such as stilbenes and anthocyanins [54, 57, 58], which agrees well with the up-regulation of stilbene synthases and anthocyanin glucosyltransferases genes in berries in this study (Fig. 5).

### ABA effects on energy conservation and antioxidant defense during water deficit

ABA is a defense hormone that helps protect plants from water deficits. Plants are very sensitive to water deficits and maintaining a sufficient level of energy is difficult when stomata are closed and photosynthesis is inhibited. Under energy-limited conditions, it has been suggested that plants prioritize metabolic pathways that support energy conservation, by reducing growth and protein synthesis, and utilizing the conserved energy for defense, such as antioxidant defenses [37].

As a consequence of energy conservation during stress, metabolic flux can be diverted from cell wall production, an energy intensive process. Here, the decreasing transcript abundance of *VviFLA11* found only in the shoot tips by the ABA treatment may be associated with the inhibition of shoot growth. A previous study showed that the *fla1* mutant has a reduced ability to regenerate shoots in an in vitro shoot-induction assay in *Arabidopsis* [59]. *AtFLA11* and *AtFLA12* in *Arabidopsis* are involved in cell wall composition, resulting in changes in cell-wall architecture [60]. This case also occurs in leaves, where a gene involved in GA biosynthesis was decreased in transcript abundance. GA regulates the transition from cell division to expansion that controls organ growth and size. Therefore, GA levels may be decreased under stress to limit the leaf area surface, which aids the plant by reducing water loss [61]. Additionally, the transcript abundance of other cell wall enzymes were affected such as a pectinesterase in the root and cell wall peroxidases in cell culture.

Overexpressing genes involved in flavonol and anthocyanin biosynthesis increase the stress tolerance of plants [62]. Furthermore it appears that different organs (roots and shoots) of maize produce different forms of terpenoid antioxidants in response to water deficit, with the root-specific terpenoids conferring drought tolerance to the plants [63]. Anthocyanin biosynthesis has been found to be affected by ABA, which plays a role in antioxidant and UV-B protection [64, 65]. In addition, terpenes in the leaves increase in response to abiotic stress and can act as defense or protective responses [66].

Consistent with these observations, ABA increased the transcript abundance of genes involved in antioxidant defense in our study. There was a differential expression of genes involved in both flavonoid and terpenoid metabolism. Different steps in flavonoid biosynthesis were affected in the different organs (Fig. 5). The transcript abundance of (E,E)-alpha-farnesene synthase genes (*TPS47*) was increased in the leaf by ABA. (E,E)-alpha-farnesene synthase is categorized in the *TPS-b* family, which can produce monoterpenes in Cabernet Sauvignon [67]. Terpenes can act as antioxidants during abiotic stress, for example, by transfer of hydrogen or electron and quenching of singlet oxygen [68].

### ABA effects on transcription

Multiple TFs are triggered by ABA to initiate or inhibit signaling downstream. In our study, TFs that had the largest change in transcript abundance belonged to the AP2/ERF superfamily (Fig. 4). DREB is a subfamily of AP2/ERF TFs that regulate the response of plants to abiotic stress conditions [69, 70]. AP2/ERF TFs can crosstalk with other hormones such as ethylene, gibberellin, and salicylic acid. Little is known about which particular DREBs act as a sensor in each case. Our results showed a unique and high expression of the *VviDDF2* gene in shoot tips, *VviDREB2H* specifically in roots, and *VviERF022* in cell culture in response to ABA. Overexpression of the *AtDDF2* in *Arabidopsis* results in a dwarf phenotype, on account of a reduction of GA, which is consistent with our results in leaves where genes involved in GA biosynthesis were down regulated in response to ABA [71].

Other TFs families such as bZIP/ABRE, NAC, and MYC/MYB also have a differential change in gene expression. Transcript abundance of the *VviMYB121* uniquely increased in roots in response to ABA. This result is consistent with an *Arabidopsis* study where expression of *AtMYB121* increased in response to ABA treatment and only in roots; it also showed the greatest change in transcript abundance in response to osmotic stress [72]. In leaves, transcript abundance of a *VviNAC1* had the highest increase in transcript abundance of any TF in response to ABA. The ortholog of this gene in *Arabidopsis* only shows a high expression in senescent leaves [73], and some NAC TFs have been found to be part of the ABA induction of leaf senescence [74]. *VviNAC1* responds to ABA and other defense-related hormones; it confers stress tolerance when overexpressed in Arabidopsis [75]. Interestingly, the TF that had the highest transcript abundance in berries was *VviABI3*. The close ortholog of this gene in *Arabidopsis* is found to be essential for seed maturation and is part of the ABA signaling network [31]. These TFs are good candidate genes to study the regulation of organ-specific responses to ABA in grapevine.

### ABA effects on protein modification

Genes involved in protein modification and metabolism were affected by ABA in our study. Posttranslational modifications such as phosphorylation, ubiquitination, and nitrosylation have been found to be involved in ABA signaling pathways [6]. Phosphorylation is a key process for ABA to trigger downstream targets. PP2C is a key protein that negatively regulates SnRK2s via dephosphorylation. When SnRK2s are phosphorylated they are active, resulting in the phosphorylation of downstream activator and repressor proteins. PP2Cs had increased transcript abundance to slowdown the activation of the ABA signaling pathway that occurs from a rapid increase in the amount of exogenously applied ABA. These transcripts fall within the GO category for protein amino acid phosphorylation process that was found to decrease in shoot tips, roots and cell cultures (Additional file 4).

Ubiquitination is a rapid posttranslational modification that responds to various environmental stresses leading to protein degradation. In our study, we found a large increase in transcript abundance of a Plant U-box domain-containing protein 19 (PUB19) in cell culture and roots in response to ABA. A previous study found that *AtPUB19* (AT1G60190.1) gene expression was induced by ABA and drought and it has E3 ligase activity [76]. PUB19 is a negative regulator in ABA-mediated drought stress response; the *atpub19* mutant showed more sensitivity to ABA with enhanced tolerance to drought stress [77].

## Conclusions

Our transcriptomic analysis has revealed unique effects of ABA in different grapevine organs. Supporting our hypothesis that the response to a mild level of ABA was complex and dependent on the organ involved. While there was not a common response to ABA for any gene in all organs, there were common pathways or gene ontologies that were affected by applied ABA, including transcription factor activities, ABA metabolism and signaling, and flavonoid metabolism. This study provides ABA-responsive candidate genes in each grapevine organ. Identifying the differences in gene expression that regulate grapevine ABA responses in individual organs is crucial for fully understanding hormone action and the physiological responses to water deficit in the whole plant. Ultimately this knowledge can be utilized to manipulate the effects of ABA in different organs to reach desirable outcomes such as enhanced drought tolerance and grape quality.

## Methods

### Sample collection

Shoot tips and berries samples were collected at the University of California, Davis in 2010; cell culture samples were sampled at Oregon State University in 2010; roots and leaf samples were collected at the University of Nevada, Reno in 2011.

Own-rooted vines of *Vitis vinifera* (L.) cv. Cabernet Sauvignon were used for the shoot tips and berries (before véraison) assays at UC Davis. These vines were grown from dormancy in 4-L tree pots filled with 1/3 peat, 1/3 sand, 1/3 redwood compost, with 2.4 kg m^−2^ dolomite lime in a greenhouse (30/20 °C ± 3 °C; 40/70 % ± 10 % RH; and natural light with a daily maximum of 1200 μmol m^−2^ s^−1^ PAR). The vines were pruned to two shoots, and the shoots were vertically trained to ~1.5 m. Pots were drip irrigated four times a day (at 06.00, 09.00, 14.00, and 18.00) for 4 min at 7.57 L h^−1^ (2 L d^−1^) with dilute nutrient solution (90 ppm calcium, 24 ppm magnesium, 124 ppm potassium, 6 ppm nitrogen as ammonium, 96 ppm nitrogen as nitrate, 26 ppm phosphate, 16 ppm sulfate, 1.6 ppm iron, 0.27 ppm manganese, 0.16 ppm copper, 0.12 ppm zinc, 0.26 ppm boron, and 0.016 ppm molybdenum) at pH 5.5 to 6.0.

Cell suspension cultures (CS4) at Oregon State University were maintained under continuous fluorescent light (~68 μmol m^−2^ s^−1^) at 25 °C on an orbital shaker (120 rpm). Suspension cultures were subcultured weekly in 250 mL Erlenmeyer flasks containing 50 mL of cell suspension in B5 medium supplemented with 20 g L^−1^sucrose, 250 mg L^−1^ casein hydrolysate, 0.5 mg L^−1^1-naphtalene-acetic acid and 0.12 mg L^−1^ benzylaminopurine, by inoculating the cells at a 1/5 (v/v) ratio into a fresh medium. For experimental purposes, 7-day-old cell suspensions were inoculated into a fresh medium (3:7) and cultured for 3 days before treatment.

Young vines propagated from leaf cuttings were grown in an aeroponic system in a greenhouse at UNR. The rooted cuttings from Cabernet Sauvignon were grown in a growth chamber for 2 to 3 weeks before being carefully transferred to the aeroponic system. Each container (43.2 cm (L) × 27.9 cm (W) × 20.3 cm (H)) had its own aeroponic nebulizer with a fogger head size of 3.8 cm diameter × 4.4 cm height for each experimental replicate (three containers for control and three containers for ABA treatment). There were small holes in the lid of each container large enough for the rooted plant to pass through. Gibeaut’s solution [78] was used to provide the macronutrients and micronutrients to the vines in the aeroponic mist. The pH of the solution was maintained at 6.0. Root and leaf samples were grown for 3 months in this system before treatment.

### ABA treatment

A 10 μM ABA spray was made by first dissolving ABA ([+]-ABA, A.G. Scientific, Inc., http://www.agscientific.com) to 500 mM in 100 % ethanol and then diluting to 10 μM in water containing 0.05 % adjuvant (Latron-B, DOW AgroSciences LLC, http://www.dowagro.com). Control solution spray was distilled-deionized water containing 0.05 % adjuvant and 0.002 % ethanol. Shoot tips, berry clusters, and cell culture were sprayed with the 10 μM ABA treatment until running off. Roots were treated with 10 μM ABA in the aeroponic system by adding the ABA to the nutrient solution surrounding the nebulizer for 2 h; roots and leaves of root-treated vines were harvested. Samples were quickly rinsed and rapidly frozen in liquid nitrogen before storage at -80 °C. Three independent experimental (and biological) replicates were harvested to compare between the ABA-treated and untreated samples.

### RNA extraction and microarrays

Total RNA was isolated using a cetyltrimethylammonium bromide (CTAB) based method [79] and RNeasy plant mini kit (Quiagen) following the manufacture’s protocol. The total RNA was treated with RNAse-free DNAse I (Qiagen) to eliminate any genomic DNA contamination and then quantified by using a Nanodrop spectrophotometer (Thermo Scientific NanoDrop 2000c). An aliquot of each RNA sample was also analyzed using an Agilent 2100 Bioanalyzer using RNA LabChip® assays according to the manufacturer’s instructions. NimbleGen whole genome microarrays (090818 Vitis exp HX12 (Roche, NimbleGen Inc., Madison, WI, USA)) containing 29,550 probesets for genes of *Vitis vinifera* were used to determine gene expression. Total RNA (10 μg) was used to prepare cDNA using the SuperScript III First Strand System (Invitrogen), and the 2^nd^ strand was generated using random primers and klenow exo-enzyme (NEB). The cDNA was analyzed and quantified using the Bioanalyzer. Sample labeling was performed using the NimbleGen One-Color DNA Labeling Kit (NimbleGen) by following the manufacturer’s protocol. The purified labeled RNA was quantified by spectrophotometry as mentioned previously. A 3-μg sample of labeled RNA was hybridized to the array according to the manufacturer’s recommendations.

### Analysis of microarray data

All NimbleGen custom oligonucleotide array images were first examined visually in their raw data format for gross spatial variation due to fibers or bubbles. All raw array data were processed and normalized first by Robust Multi-Array Average (RMA) [80] using the R package affy [81]. Specifically, expression values were computed by applying the RMA model of probe-specific correction of perfect match probes. The processed probe values were then normalized via quantile normalization, and a median polish was applied to compute one expression measure from all probe values. The three expression measurements for each biological state were inspected individually for each element on the array as in [82]. An additional quality control step was performed at this point [83–85].

Of the 88,650 sets of replicates, 14 % exhibited a coefficient of variation greater than 50 %. Any set of replicates that displayed a coefficient of variation of greater than 0.5, and that included one or more replicated measures lying more than 1 standard deviation away from the mean was scrutinized. The maximum standard deviation for three replicates in this dataset was 1.15; 33 % of all measurements observed were greater than 1 standard deviation away from the mean of their set of replicates. Of the 886,500 expression values measured in this experiment, 11,671 (1.31 %) were excluded as single outliers. Upon this correction, there remained 171 sets of triplicates that still had coefficients of variation that were still greater than 0.75, which were completely excluded from further analysis. The remaining 874,316 (98.6 %) values had an average coefficient of variation of 0.157, which is less than typical of the microarray experiments processed by the UNR Center for Bioinformatics [83–85].

A simple two-way ANOVA was performed on the cleansed normalized data to examine probesets with statistically significant treatment effects, organ effects, and treatment and organ interaction effects. A multiple testing correction was applied to adjust the *p*-values of the ANOVA [86]. The Tukey post-hoc test was applied for the genes with a significant treatment and treatment x organ interaction terms (adjusted *p*-value ≤ 0.05). The genes that were statistically significantly different from the post-hoc test were used to create a Venn diagram in Fig. 1b.

Principal component analysis (PCA) was applied to quality-controlled expression data using the covariance matrix to visualize any trends in the expression data. Metabolic pathways affected by ABA were determined using a gene ontology (GO) file created using the EnsemblPlants BioMart [87] for *Vitis vinifera* and BiNGO analysis [88] (v. 2.44, http://apps.cytoscape.org/apps/bingo) with Cytoscape [89] (v. 2.8.3, http://www.cytoscape.org/). Overrepresented categories were determined using a hypergeometric test with a significance threshold at 0.05 after a Benjamini and Hochberg false discovery rate correction.

### qPCR

Array verification was performed by qPCR for 10 differentially expressed genes (Additional file 6). A coefficient of determination (r^2^) between microarray and qPCR was 0.69, *P* < 0.0001. Total RNA extraction was the same as that used for microarray analysis described above. One μg of RNA was reverse transcribed to cDNA using the iScript reverse transcriptase supermix (Bio-Rad) according to manufacturer’s instructions. Quantitative real-time RT-PCR (qPCR) experiments were conducted using SsoAdvanced SYBR Green Supermix (Bio-Rad). The PCR primers were designed using the NCBI Primer-blast software. The primer sequences and PCR efficiency were provided in Additional file 6. Reactions were carried out on a BioRad Real-time thermal cycler CFX96. The two-step thermal cycling was used for all PCR experiments: 95 °C for 30 s; 40 cycles of 95 °C for 10 s, 59 °C for 15 s and 95 °C for 10 s. Fluorescence signals were captured at the end of each cycle, and the melting curve analysis was performed from 53 °C to 95 °C to determine the specificity of the amplified products. A reference gene was selected from genes that had a lower coefficient of variation in all experiments using the NimbleGen grape whole-genome microarray 29 K 090918-MD. *GAPDH* [EnsemblPlants:VIT_17s0000g10430], *EIF4F* [EnsemblPlants:VIT_04s0008g06770], and *EF1alpha* [EnsemblPlants:VIT_06s0004g03220] were tested for suitable and reliable reference genes for this experiment. A *GAPDH* gene was chosen in the assays as an internal control for normalizing the variations in all data due to a low coefficient of variation of mRNA through microarray analysis. The relative expression ratio value was calculated according to the Pfaffl equation [90].

### Availability of supporting data

All microarray expression data are available at the Gene Expression Omnibus (GEO) database [91] with the accession number GSE78798.

## Additional files

Additional file 1: Annotation, transcript abundance values, and statistics of all genes on the NimbleGen Grape Whole-Genome microarray for the different organs of Cabernet Sauvignon. Adj means *p*-values after FDR correction at the 0.05 level. Transcript values are the mean log_2_ values of each probe set for each treatment. (XLS 16032 kb) Additional file 2: Significant genes from 2-way ANOVA (AdjTrt and AdjTrt*Organ with *p* ≤ 0.05) were further tested by the Tukey Post-Hoc test. Values are the probability from the Tukey Post-Hoc test and log_2_ratio of ABA treated transcripts divided by control treated transcripts for each organ. Significant down-regulated genes and up-regulated genes from the Post-Hoc test were highlighted in blue and red, respectively. (XLS 353 kb) Additional file 3: The profiles of the log_2_ ratio (ABA/Control) of the transcript abundance of the genes (probesets) involve in ABA biosynthesis, degradation & conjugation, transport, ABA signaling, transcription factors, and flavonoid biosynthesis. (XLS 59 kb) Additional file 4: BiNGO results for overrespresented GO biological process functional categories of all significant genes, down-regulated genes in shoot tips and roots, and up-regulated genes in roots and cell cultures from the Tukey Post-Hoc test (*p* ≤ 0.05). (XLS 1139 kb) Additional file 5: Validation of microarray data by qPCR and the primer sequences and primer efficiency of reference genes and target genes. (XLS 188 kb) Additional file 6: Array verification by qPCR for 10 differentially expressed genes. Scatter plots are shown for a correlation between gene expression measured by microarray (y axis) and qPCR (x axis) for 10 differentially expressed genes. Log_2_ of fold change between treatment and control was plotted. Pearson’s correlation test: *R* ^*2*^ *=* 0.6868, *P <* 0.0001. (XLS 165 kb)
